# Biological Degradation of Chinese Fir with *Trametes Versicolor* (L.) Lloyd

**DOI:** 10.3390/ma10070834

**Published:** 2017-07-20

**Authors:** Meiling Chen, Chuangui Wang, Benhua Fei, Xinxin Ma, Bo Zhang, Shuangyan Zhang, Anmin Huang

**Affiliations:** 1 International Centre for Bamboo and Rattan, Beijing 100102, China; meiling1226@163.com (M.C.); maxx@icbr.ac.cn (X.M.); 2 Anhui Agricultural University, Hefei 230036, China; zsyhj_2006@163.com; 3 Qingdao Institute of Bioenergy and Bioprocess Technology, Chinese Academy of Sciences, Qingdao 266101, China; boews@sina.com; 4 Chinese Academy of Forestry, Beijing 100091, China; hbham2000@sina.com

**Keywords:** Chinese fir, white-rot fungus, mechanical properties, chemical composition, crystallinity

## Abstract

Chinese fir (*Cunninghamia lanceolata* (Lamb.) Hook.) has been an important afforestation species in northeast China. It has obvious defects of buckling and cracking easily, which are caused by its chemical components. *Trametes versicolor* (L.) Lloyd, a white-rot fungus, can decompose the cellulose, hemicellulose, and lignin in the wood. White-rot fungus was used to biologically degrade Chinese fir wood. The effects of different degradation time on the Chinese fir wood’s mechanical properties, micromorphology, chemical components, and crystallinity were studied. The results showed that the heartwood of Chinese fir was more durable than the sapwood and the durability class of Chinese fir was III. *Trametes versicolor* (L.) Lloyd had a greater influence on the mechanical properties (especially with respect to the modulus of elasticity (MOE)) for the sapwood. *Trametes versicolor* (L.) Lloyd degraded Chinese fir and colonized the lumen of various wood cell types in Chinese fir, penetrated cell walls via pits, caused erosion troughs and bore holes, and removed all cell layers. The ability of white-rot fungus to change the chemical composition mass fraction for Chinese fir was: hemicellulose > lignin > cellulose. The durability of the chemical compositions was: lignin > cellulose > hemicellulose. The crystallinity of the cellulose decreased and the mean size of the ordered (crystalline) domains increased after being treated by white-rot fungus.

## 1. Introduction

Wood cell walls are the load-bearing unit in trees and can be regarded as laminated nanocomposites, in which cellulose microfibrils are embedded in the matrix of hemicellulose and the lignin is a reinforcement. The wood cell wall mainly consists of cellulose, hemicellulose, and lignin. The interaction of chemical components and their mechanical properties leads to specific mechanical properties of wood cell walls and gradually affect the macroscopic properties of wood. The future use of the wood is determined by the wood’s properties. Therefore, a deep understanding of the structure–property relationship is crucial for understanding the nature origin of the physical and mechanical properties of wood. A better understanding of the properties could give more indications of how to make full use of the wood.

To explore the relationship between wood properties and chemical compositions, there has been a wealth of research conducted on removing the chemical components from wood cell walls through a chemical method. Moreover, the chemical composition of wood is found to have a significant influence on the mechanical properties [1,2,3]. It is also found that a decline in hemicellulose affects the integrity of the cell wall polymers and decreases the strength against mechanical loads. However, there are few approaches of biological natural degradation to remove chemical composition in wood cell walls. White-rot fungus, *Trametes versicolor* (L.) Lloyd, has an efficient ability to degrade cellulose, hemicellulose, and lignin [4,5,6,7]. Perez et al. [8], Davis et al. [9], Shangguan et al. [10], and Bari E et al. [11,12] came to the same conclusions and reported that the white-rot fungus can attack both cellulose and lignin. The lignin component of the sample was intensely degraded by white-rot fungus and the strength of sample was decreased [10,12]. Above all, the white-rot fungus was used in this research to remove chemical compositions and to explore the relationship between the chemical compositions and mechanical properties of wood. Therefore, a comprehensive analysis of chemical constituents and wood properties can be obtained when the wood is biologically, naturally degraded by white-rot fungus.

*Cunninghamia lanceolata* (Lamb.) Hook. (Chinese fir), an important afforestation species in northeast China, is well known for providing the most abundant wood materials in China [13,14]. The area of planted Chinese fir forest has reached 69 million hm^2^ [13]. Chinese fir is widely used in some fields, such as in constructing buildings, bridges, furniture, and so on. However, the properties of planted Chinese fir wood are less than satisfactory and have obvious defects caused by buckling and cracking easily. A deeper understanding of the mechanical properties of Chinese fir, particularly the aspect of cell wall chemical compositions, can help to make full use of the Chinese fir wood with significant benefits.

In this paper, the biological degradation of fir wood and its influence on the wood properties were investigated. For this purpose, the biological degradation capabilities during different decay processes, physical and mechanical properties, as well as the changes of chemical compositions were compared to evaluate the similarities and differences between different portions. Changes of physical, chemical, and mechanical properties were evaluated at six weeks, 12 weeks, and 18 weeks. The objective was to explore the relationship between the chemical compositions and mechanical properties of the wood, in order to make full use of the Chinese fir wood.

## 2. Results

### 2.1. Mass Loss

**Figure 1:**
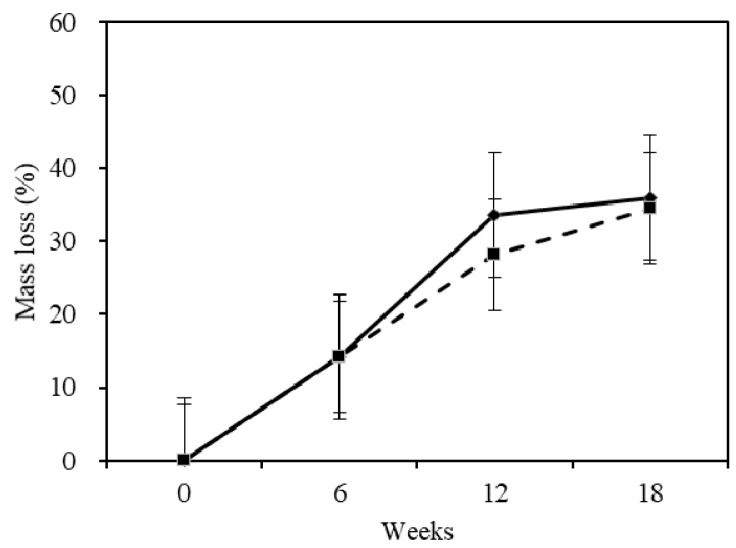
Mass loss of samples after degradation. The continuous and discontinuous lines in the figure caption indicates sapwood and heartwood. The error bar is according to a confidence interval of 95%.

The relationship between wood weight loss and decay time was determined by measuring the average weight losses of 20 to 30 test specimens at a six-week interval over an 18-week decay period. The mass loss of sapwood and heartwood with three time points are shown in Figure 1. The mass loss of sapwood and heartwood was 33.6% and 28.2% at 12 weeks, respectively (Figure 1), which was based on the results of the species of fungus that caused the highest average wood weight loss in the test specimens. The heartwood was more durable than the sapwood. According to the classification of the natural durability of wood (Table 1), sapwood and heartwood were both slightly durable to the white-rot by *Trametes versicolor* (L.) Lloyd; the durability class was III. It can be seen that the mass loss for the sapwood and heartwood were similar during the whole degradation and reached 36.0% and 34.5% at the end of 18 weeks, respectively (Figure 1). The slopes of the two samples were similar, which indicates that the fungi caused similar degradation rates during the 18-week exposure period under the conditions of this study.

### 2.2. Mechanical Properties

As fungi grew through the wood, it modified the chemical structure and removed cell wall constituents, thereby changing the mechanical properties [16]. Highly-oriented cellulose microfibrils and the encrusting hemicellulose and lignin contribute to the strength of the wood. Therefore, any changes in these components caused by decay fungi often result in sharp reductions in wood strength [17].

**Figure 2:**
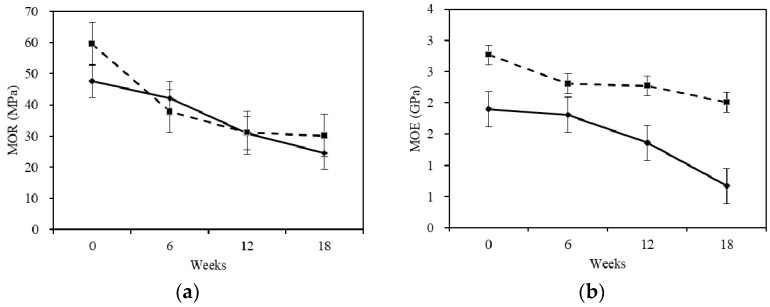
Changes of (**a**) modulus of rupture (MOR) and (**b**) modulus of elongation (MOE) before and after degradation. The continuous and discontinuous lines in the figure caption indicates sapwood and heartwood. The error bar is according to a confidence interval of 95%.

Three stages were divided by six-week intervals in this study, as shown in Figure 2. The mechanical properties of the sapwood and heartwood shared decreasing trends. Little difference in the modulus of rupture (MOR) between sapwood and heartwood in the same stage was consistent with the minimal difference in cellulose, which provides strength to the wood. A greater difference in the modulus of elasticity (MOE) between the sapwood and heartwood is consistent with the greater loss of hemicellulose and lignin (which gives toughness to the wood) in sapwood than in heartwood. For the heartwood, the MOR and MOE showed reductions in the first stage. The decrease of MOR and MOE seemed to be smaller in the second stage than in the last stage, respectively. For the sapwood, the MOR and MOE continued to decrease in the overall degrading period. The values decreased slowly at the first stage, while rapidly decreasing in the second and last stages. The MOE of the heartwood was still higher than the MOE of the sapwood exposed to white-rot fungus during the whole stage. This was because the more durable heartwood exhibited less mass loss and supported higher mechanical properties. For the heartwood, the total reduction of MOE was 27.5% and that of MOR was 49.5%. For the sapwood, the total reduction of MOE was 64.7% and that of MOR was 48.7%. The reduction rate of MOE for the sapwood was 3.6 times that of the heartwood, while the reduction rate of MOR was similar with heartwood. The results elucidated that the white-rot fungus had a greater influence on the mechanical properties (especially with respect to the modulus of elasticity (MOE)) for the sapwood.

### 2.3. Characterization

**Figure 3:**
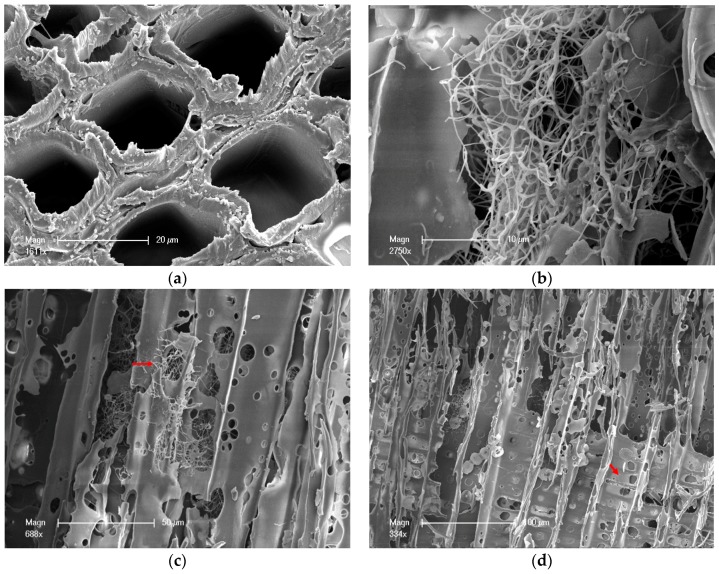

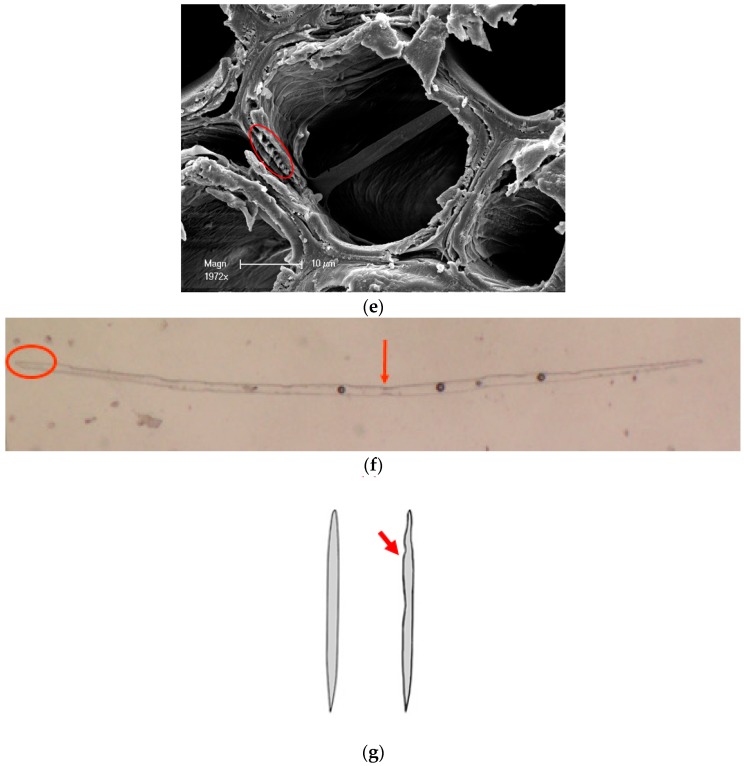
(**a**) Untreated wood samples; (**b**) Hyphae spread in the wood cells; (**c**) Hyphae spread by penetrating pit; (**d**) The cell wall became thinner after degradation, the arrow shows the area becoming thinner; (**e**) Treated wood samples; (**f**) The treated tracheid; (**g**) The change of tracheid before and after degradation. The surface of untreated tracheid (left) was smooth and the end of it was spinous. The end of the tracheid and the middle of treated tracheid (right) surface became rough.

Figure 3a shows the untreated wood. In the white-rot fungus infection, the mycelium spread in all forms of cells during the whole degradation (Figure 3b), especially in the resin and wood ray cells [18]. Hyphae spread in the wood mainly by penetrating pits, although they can also penetrate the cell wall directly (Figure 3c). The mycelium close to the surface of the cell walls was clothed with a glucan sheath and released a variety of enzymes [19]. Thus, these enzymes gradually digested the cell walls and produced monosaccharide nutrients [19]. In addition to enzymes acting directly, the fungal conversion and attack on lignocellulose progresses at a distance from the hyphae with the help of small diffusible oxidants and secreted metabolites. Usually, the digested monosaccharide was absorbed through the sticky sheath by the hyphae. Hyphae in the wood did not enter cells’ corners, but only gradually cut cell walls thinner through the sticky sheath close to the cell wall surface (Figure 3d). When the Chinese fir wood was eroded by white-rot fungus (Figure 3e), the cell corner erosion [20] led to the reduction of the MOE and MOR. Scanning electron microscopy (SEM) revealed that the white-rot fungus colonized the lumen of various wood cell types in Chinese fir, penetrated cell walls via pits, caused erosion troughs and bore holes, and removed all cell layers [16].

After the wood was degraded by white-rot fungus, the tracheid form of wood changed correspondingly (Figure 3f,g). The end and the middle parts of the tracheid became rough, as shown by the arrow marked. It decreased the connectivity of wood tracheid and resulted in the reduction of the mechanical properties.

### 2.4. Chemical Properties Analysis

#### 2.4.1. Chemical Compositions

**Figure 4:**
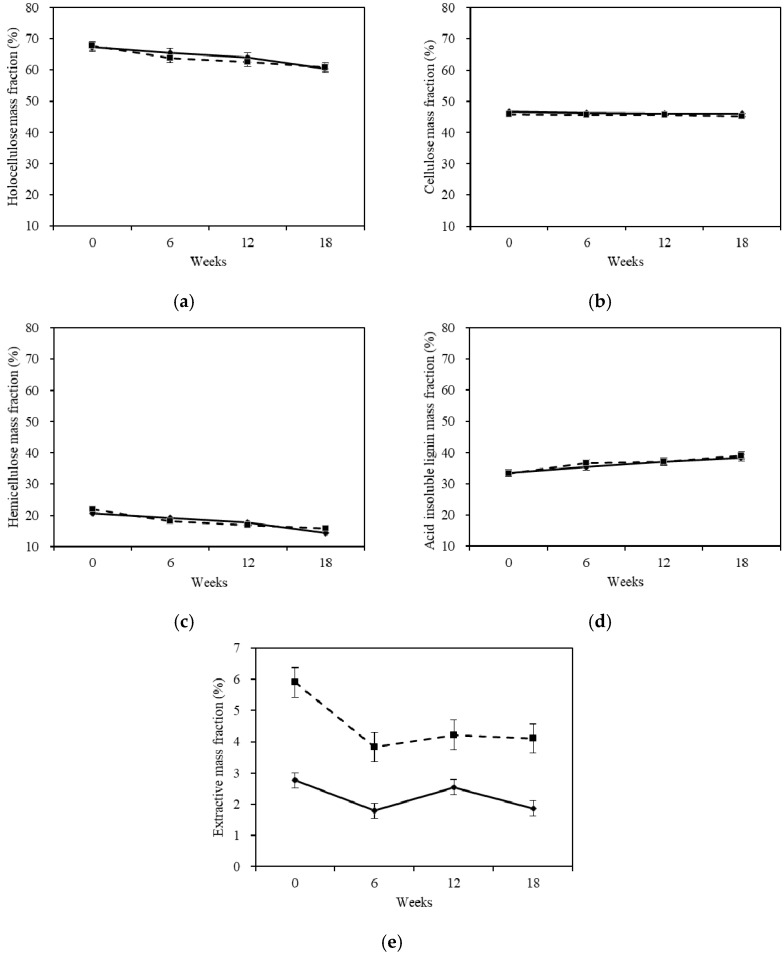
Changes of chemical compositions mass fraction after degradation. (**a**) Changes of holocellulose mass fraction; (**b**) Changes of cellulose mass fraction; (**c**) Changes of hemicellulose mass fraction; (**d**) Changes of acid-insoluble lignin mass fraction; (**e**) Changes of extractives mass fraction. The continuous and discontinuous lines in the figure caption indicates sapwood and heartwood. The error bar is according to a confidence interval of 95%.

Figure 4 shows the changes of the Chinese fir wood chemical compositions mass fraction after being degraded by white-rot fungus. Changes in the chemical compositions of Chinese fir during decay by the white-rot fungus in different stages are presented in Table 2. The chemical compositions are expressed as a percentage of the weight of the original oven-dried untreated wood specimens. The percentage losses for the compositions by the fungus during decay are presented in Table 3, expressed as a percentage of the appropriate value for the oven-dried untreated wood.

The mass fraction of holocellulose, cellulose, and hemicellulose decreased, while acid-insoluble lignin increased during the whole degradation. The changes of the chemical composition mass fractions were similar to that of *Betula luminifera* after being treated by white-rot fungus (*Corliolus versicolor* (L.) Quél) [21]. However, these were still some notable differences.

It can be observed in Figure 4a that holocellulose, the sum of hemicellulose and cellulose, gradually decreased in sapwood and heartwood with increasing exposure time. Moreover, the change trends of sapwood and heartwood were similar. The fungi caused a significant decrease in the holocellulose mass fraction of heartwood in stage 1, and the degrading effect for heartwood was remarkably higher in this period. As the decay process continued, the holocellulose mass fraction reduction of the sapwood and heartwood became similar. The holocellulose mass fraction reached a similar decreasing trend around 61% in stage 3. From Table 2, it can be seen that the holocellulose of Chinese fir decreased during the whole infection. Comparing the holocellulose degradation speed between sapwood and heartwood, according to Table 3, the heartwood had a higher speed in stages 1 and 3, while the sapwood had a higher speed in stage 2.

The cellulose mass fraction of sapwood and heartwood did not show any dramatic changes in Figure 4b. The cellulose mass fraction of heartwood decreased slightly during the period of 18 weeks, whereas the sapwood cellulose mass fraction only decreased apparently in stage 1, and no significant difference was observed from stage 2 to stage 3. These changes were very small. However, when celluloses were expressed as a percentage of the weight of the original oven-dried untreated wood specimens, it can be seen that the celluloses decreased during the whole degradation time (Table 2). The decline of mechanical properties can also clarify the loss of cellulose. Combining with the mass loss data analysis, it can be inferred that the white-rot fungus can degrade the cellulose, but might not change the mass fraction of cellulose in the wood. As shown in Table 3, the cellulose percentage loss between sapwood and heartwood in stage 1 was similar, which demonstrated that the cellulose degradation speeds of the two specimens were similar in this stage. The cellulose percentage loss of sapwood was higher than heartwood in stage 2, which indicated that the sapwood had an obviously higher degradation speed during this period. During the last stage, the cellulose degradation speed of heartwood was quicker than the sapwood from the percentage loss data.

The change in hemicellulose mass fraction was similar to the total carbohydrates, as shown in Figure 4c. The hemicellulose mass fraction of sapwood and heartwood both had decreasing trends. The white-rot fungus steadily degraded the hemicellulose. The decline in hemicellulose mass fraction affected the integrity of the cell wall polymers and decreased the strength against mechanical loads [22]. The hemicellulose of sapwood and heartwood decreased by 30.8% and 28.0% during the whole cultivation period. During the first two stages, the white-rot fungus had a more evident effect on heartwood’s hemicellulose. The hemicellulose of heartwood had a higher percentage loss and degradation speed. In the last stage, the hemicellulose of sapwood had a higher percentage loss and degradation speed.

As is observed in Figure 4d, both samples showed a steady increase in the acid-insoluble lignin mass fraction during the 18 weeks. Judging from the changes, the white-rot fungus can change the acid-insoluble lignin mass fraction in the sapwood and heartwood. The acid-insoluble lignin mass fraction of sapwood and heartwood increased 5.9% and 5.7% during the whole cultivation period, respectively. Even though the acid-insoluble lignin mass fraction of sapwood and heartwood increased during the degradation time, there was still a decline of lignin mass, as shown in Table 2. The mass loss analysis and the decline of mechanical properties also demonstrated the decline of lignin. Lignin in wood supplies a physical obstacle to enzymatic disintegration of cellulose and hemicellulose [23]. However, the holocellulose of both the sapwood and heartwood underwent obvious declines, as shown in Table 1, indicating a poor ability of the lignin to prevent the white-rot fungus from degrading the holocellulose. When comparing the acid-insoluble lignin percentage loss between the sapwood and heartwood, it was found that the white-rot fungus had a strong ability to degrade the sapwood’s lignin and the degradation speed of sapwood was higher than heartwood.

Extractives are the most important factors contributing to the natural durability of wood, although many other factors may also influence wood biodegradability [17,24,25]. In this study, heartwood was proven to be more durable than sapwood because its extractive mass fraction was higher, at 5.9%. These results are in agreement with the results of other researchers, who have shown that wood species in which the extractive content is over 6% have high durability against decay by fungi [26]. The total extractives mass fraction decreased, as shown in Figure 4e. The results showed that the degradation rate of the wood macromolecular components was lower than that of the consumption of these degradation products. The degradation products did not accumulate in the treated wood, and the fungi also consumed many extractives that were originally dissolved in benzene [27,28,29]. Comparing the first two stages, it can be found that both of the samples’ extractives mass fraction presented an increasing trend. It was possible that the rate of producing the degradation products was higher than the consumption. In the last stage, the extractives mass fraction of sapwood and heartwood decreased slightly. Above all, the variation trends of the extractives mass fraction for sapwood and heartwood were similar to each other.

In summary, the ability of white-rot fungus to change the chemical composition mass fraction for sapwood and heartwood was: hemicellulose > lignin > cellulose. The durability of chemical compositions was: lignin > cellulose > hemicellulose.

#### 2.4.2. X-ray Diffraction

**Figure 5:**
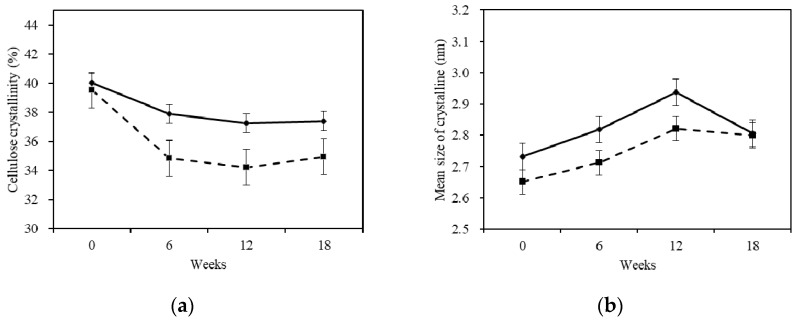
Changes of (**a**) cellulose crystallinity and (**b**) crystalline mean size before and after degradation. The continuous and discontinuous lines in the figure caption indicates sapwood and heartwood. The error bar is according to a confidence interval of 95%.

With the increase of degradation time, the crystallinity of cellulose declined gradually (Figure 5). The cellulose crystallinity of sapwood was also reduced. The decreasing speed of sapwood cellulose crystallinity was steady. In the first stage, the decreasing speed of cellulose crystallinity was relatively higher, with a total decrease of 5.3%. The cellulose crystallinity was decreased by 6.9% in the second stage and 6.5% in the third stage. The cellulose crystallinity of heartwood was decreased in the first stage by 11.8%, and by 13.4% during the second stage. However, the crystallinity increased in the third stage, probably due to the rearrangement of the cellulose hydrogen bond and the effect of recrystallization. In general, the crystallinity of Chinese fir sapwood cellulose had a similar variation trend to the sapwood, after being infected by white-rot fungus. The cellulose crystallinity of Chinese fir had a similar decreasing trend, which resembled the phenomenon when *Betula luminifera* was treated by white-rot fungus (*Corliolus versicolor* (L.) Quél) [21].

The mean size of crystalline in both of the samples was increased after being degraded by white-rot fungus. It is possible that large amounts of hemicellulose were degraded by white-rot fungus, leading to the gathering of cellulose and the subsequent increment of the mean size of crystals.

## 3. Materials and Methods

### 3.1. Materials

**Figure 6:**
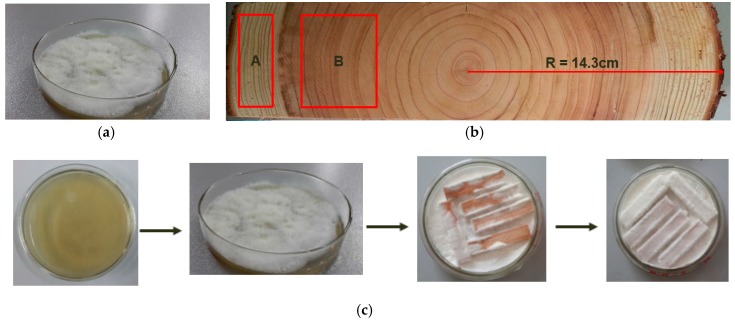
(**a**) Fungal pure cultures were held on the potato dextrose agar; (**b**) Wood samples and sampling areas; (**c**) Treated sample preparation.

The white-rot fungus was collected and purified at the Research Institute of Forest Ecology Environment and Protection, Chinese Academy of Forestry (CAF). The white-rot fungus used in this study was directly bought from CAF. Fungal pure cultures were held on the potato dextrose agar (PDA) in Petri dishes in accordance with Huang [16] (Figure 6a). Wood disks were cut from two 37-year-old Chinese firs (*Cunninghamia lanceolata* (Lamb.) Hook.) at breast height. The diameter of the disk was 28.6 cm. The thickness of the disks was 10 cm–15 cm, as shown in Figure 6b. The sapwood and heartwood were distinguished by their color. The areas A and B in Figure 6b show the sapwood and heartwood used in this study, respectively. The disks were air-dried to 23 ± 2% moisture content and then divided into sapwood and heartwood. Specimens with a size of 50 mm × 10 mm × 5 mm (longitudinal × radial × tangential) were prepared for the following experiments. The treated sample preparation is shown in Figure 6c. The measured data was analyzed by the Statistic Package for Social Science (SPSS 19, IBM, Chicago, IL, USA) [30].

### 3.2. Biological Degradation

Samples were dried in an oven at 103 ± 3 °C and weighed, after which sterilization was performed at 121 °C for 20 min. After completing the growth of the fungal mycelia in Petri dishes, the wood blocks were exposed to grown mycelia on PDA in Petri dishes. Conditioned specimens were incubated for six, 12, and 18 weeks at 28 °C and 78% relative humidity (RH). Mycelia were removed from the block surfaces after the incubation. The blocks were dried at 103 ± 3 °C and weighed again to calculate the mass loss (ML) according to Equation (1) (GB/T 13942.1-2009) [15]:

(1) $$\mathrm{ML}(\%)=\frac{M_{1}-M_{2}}{M_{1}}\times 100$$

where *M*_1_ (g) and *M*_2_ (g) are the dry mass before and after incubation, respectively.

The natural durability classification of the test species was examined according to GB/T 13942.1-2009 [15], as shown in Table 1. This was based on the results of the species of fungus that caused the highest average wood weight loss in the test specimens after an exposure period of 12 weeks.

### 3.3. Mechanical Test

The samples for three-point bending were tested according to ASTM-D143-94 [31] with a displacement rate of 1 mm/min. The length, width, and thickness of each specimen were recorded. Dimensions of the specimens were prepared with the following ranges: length (along the longitudinal direction), 49–50 mm, width (along the radial direction) 9–10 mm, and thickness (along the tangential direction), 3–4 mm. Span was set with the span-to-depth ratio of no less than 16 to avoid shear stresses. Ten replicates were tested in this section.

### 3.4. Characterization

#### 3.4.1. Tracheid Morphology

Blocks of 5 mm × 1 mm × 1 mm (longitudinal × radial × tangential) were placed in test tubes with the mixed solution (the ratio of 30% H_2_O_2_ and CH_3_COOH is 1:1) in the furnace (60 °C) until the tracheid turned white. The tracheid was washed with distilled water until the pH was neutral. After that, the blocks were stirred into floccule to extract single tracheid prepared for further observations. A stereomicroscope (SMZ-168-BL, Xiamen, China) was used for tracheid morphology observation.

#### 3.4.2. Environment Scanning Electron Microscope Observation

An environment scanning electron microscope (XL30 ESEM FEG, FEI Co., Hillsboro, OR, USA) was used for observation. Blocks of untreated and treated specimens were cut into pieces of 5 mm × 4 mm × 4 mm (longitudinal × radial × tangential) with three replications for each direction. The specimens were first trimmed with razor blades and then a sliding microtome. Finally, specimens were carefully placed on ESEM stubs and gold-coated by a sputter coater to obtain a thickness of about 12 nm.

### 3.5. Chemical Properties Analysis

#### 3.5.1. Chemical Compositions

Changes in the chemical compositions of wood cell walls in the untreated and the treated wood were evaluated on the basis of Chinese National Standards for carbohydrate analysis [6,32]. The treated specimens were dried, milled, and then passed through a sieve (the size of the mesh is 420 μm) to determine the mass fraction of the lignin, cellulose, extraction, and holocellulose, according to GB/T 747-2003 [33], GB/T 744-1989 [34], GB/T 2677-1994 [35], and GB/T 2677.10-1995 [36], respectively. The hemicellulose was calculated by the following equation:

(2) $$C_{\mathrm{hemi}}=C_{\mathrm{holo}}-C_{\mathrm{cell}}$$

where *C_hemi_* is the average mass fraction of hemicellulose, and *C_holo_* and *C_cell_* are the average mass fraction of holocellulose and cellulose, respectively. Two replicates were tested in this section.

#### 3.5.2. X-ray Diffraction Measurements

X-ray Diffraction Measurements measurements were performed to assess the crystalline properties of air-dried wood cell walls by an X-ray diffractometer (AV300, Panalytical Co., Amsterdam, The Netherlands). Three replicates were tested in this section. The reflection technique was used for all measurements. The incident X-ray radiation was the characteristic Cu X-ray passing through a nickel filter with a power of 30 kV and 30 mA. The cellulose crystallinity in the fir wood cell walls was calculated by the following Segal method [37,38]:

(3) $$C_{s}=\frac{I_{200}-I_{Am}}{I_{200}}\times 100$$

where *C_s_* is the crystallinity (%), *I*_200_ is the reflection intensity of (200) plane diffraction, and *I_Am_* is the intensity at the minimum near 18.5° of 2θ angle. The Segal method is simple to apply and does not require peak separation between (110) and (110) reflections.

The Scherrer equation, in X-ray diffraction and crystallography, relates the size of sub-micrometer particles, or crystallites, in a solid to the broadening of a peak in a diffraction pattern [39]. It is used to determine the size of crystal particles in the form of a powder. The Scherrer equation used to calculate the mean size of ordered (crystalline) domains can be written as:

(4) $$D=\frac{K\lambda }{\beta \cos\theta }$$

where *D* is the mean size of the ordered (crystalline) domains, which can be smaller than or equal to the grain size, and *K* is a dimensionless shape factor, with a value close to unity. The shape factor has a typical value of about 0.9, but varies with the actual shape of the crystallite. *β* is the line broadening at half the maximum intensity, after subtracting the instrumental line broadening, in radians. This quantity is also sometimes denoted as Δ(2θ). Finally, *θ* is the Bragg angle (in degrees), and *λ* is the X-ray wavelength (0.154178 nm).

## 4. Conclusions

The results in this study led to the following conclusions:

1. Chinese fir was slightly durable, with a durability class of III. The heartwood was more durable than the sapwood.
2. The MOR and MOE of fir wood decreased after the treatment with white-rot fungus and reached the minimum in the 18th week. The white-rot fungus had a greater influence on the mechanical properties (especially the MOE) for the sapwood.
3. *Trametes versicolor* (L.) Lloyd degraded Chinese fir and colonized the lumen of various wood cell types in Chinese fir, penetrated cell walls via pits, caused erosion troughs and bore holes, and removed all cell layers.
4. The ability of white-rot fungus to change the chemical composition mass fraction for Chinese fir was: hemicellulose > lignin > cellulose. The durability of the chemical compositions was: lignin > cellulose > hemicellulose.
5. The crystallinity of the cellulose was decreased and the mean size of the ordered (crystalline) domains was increased after being treated with white-rot fungus.

## Figures and Tables

**Table 1:** Classification of the natural durability of wood to fungal attack based on GB/T 13942.1-2009 [15].

Durability Class	Description	Mass Loss
I	Very durable	ML ≤ 0
II	Durable	0.11 < ML ≤ 0.24
III	Slightly durable	0.25 < ML ≤ 0.45
IV	Not durable	ML > 0.45

ML: Mass loss.

**Table 2:** Chemical compositions of Chinese fir in progressive stages of decay caused by *Trametes versicolor* (L.) Lloyd (based on the oven-dried weight of the original untreated wood).

Samples	Weeks	Holocellulose (%)	Cellulose (%)	Hemicellulose (%)	Acid-Insoluble Lignin (%)
Sapwood	0	67.3	46.7	20.6	33.6
	6	56.2	39.6	16.5	30.3
	12	42.4	30.6	11.8	24.5
	18	38.7	29.5	9.1	24.6
Heartwood	0	67.7	45.8	22.0	33.3
	6	54.7	39.2	15.6	31.4
	12	44.9	32.7	12.2	26.6
	18	39.9	29.5	10.4	25.6

**Table 3:** Percentage loss (%) of chemical compositions of Chinese fir following decay by *Trametes versicolor* (L.) Lloyd compared to individual compositions in the original oven-dried untreated wood.

Samples	Weeks	Holocellulose (%)	Cellulose (%)	Hemicellulose (%)	Acid-Insoluble Lignin (%)
Sapwood	0	0	0	0	0
	6	16.5	15.0	19.9	9.6
	12	36.9	34.4	42.7	26.9
	18	42.5	36.7	55.7	26.7
Heartwood	0	0	0	0	0
	6	19.2	14.3	29.2	5.8
	12	33.8	28.5	44.6	20.1
	18	41.1	35.4	52.9	23.4
